# Thoracolithiasis: a rare and frequently underrecognized entity – a retrospective single-center study and narrative literature review

**DOI:** 10.1186/s12893-026-03951-8

**Published:** 2026-06-15

**Authors:** Caner İşevi, Mehmet Gökhan Pirzirenli, Berk Caner Öten, Halil Kolcu, Burçin Çelik, Yasemin Büyükkarabacak

**Affiliations:** 1 Department of Thoracic Surgery, Faculty of Medicine, Ondokuz Mayıs University, Samsun, Turkey; 2 Clinic of Thoracic Surgery, Bayburt State Hospital, Bayburt, Turkey

**Keywords:** Thoracolithiasis, Pleural loose body, Non-mobile thoracolith, Pleural nodule, Video-Assisted Thoracoscopic Surgery (VATS)

## Abstract

**Objectives:**

Thoracolithiasis is a rare benign pleural condition classically described as a freely mobile, calcified intrapleural loose body. Systematic data on non-mobile thoracolithiasis and its clinical context are scarce. This study aimed to characterize the clinical, radiologic, etiologic, and geographic features of non-mobile thoracolithiasis, to explore isolated versus secondary forms, and to identify scenarios in which these lesions mimic malignant pleural nodules.

**Materials and methods:**

This retrospective, observational study included adult patients diagnosed with thoracolithiasis at a tertiary thoracic surgery center between January 2010 and December 2024. Eligible cases had radiologic and/or surgical confirmation and complete clinical and imaging data. Demographic, clinical, radiologic, and surgical variables, including asbestos exposure and coexisting thoracic disease, were collected. Patients were stratified by sex, symptom status, calcification, and isolated versus secondary thoracolithiasis. Statistical comparisons used the Mann–Whitney U test and Chi-square or Fisher’s exact test (two-tailed *p* < 0.05). A narrative review of the literature was conducted to contextualize the findings of the present cohort.

**Results:**

Twenty patients with pathologically confirmed thoracolithiasis (mean age 64.0 ± 10.5 years; 70% male; 75% with a smoking history) were included. No lesion showed evidence of mobility on available imaging review or intraoperative assessment. 60% of lesions were non-calcified, with a mean maximum diameter of 29.4 ± 17.8 mm.

Thoracolithiasis coexisted with lung cancer in 40% and with other thoracic disease in 30%; 30% were isolated. Patients with calcified lesions were older than those with non-calcified lesions (70.0 vs. 60.0 years, *p* = 0.023). Lung cancer occurred exclusively in older male smokers and was significantly associated with male sex (*p* = 0.010) and smoking history (*p* = 0.027). Isolated thoracolithiasis was more frequent in female non-smokers, and all isolated cases underwent diagnostic exploration (100% vs. 35.7%, *p* = 0.025). Asbestos-exposed patients (25%) originated exclusively from inland provinces, whereas most non-exposed patients resided in a coastal, non–asbestos-endemic region.

**Conclusion:**

Non-mobile thoracolithiasis exhibits two clinically distinct patterns: a secondary type in older male smokers with thoracic malignancy, and an isolated type in non-smoking women without additional thoracic disease. Non-mobile, often non-calcified thoracoliths in low-risk patients frequently mimic malignant pleural nodules and may lead to avoidable thoracoscopic or open exploration. Recognizing this broader spectrum of thoracolithiasis may help refine diagnostic algorithms and reduce unnecessary invasive procedures.

**Supplementary Information:**

The online version contains supplementary material available at 10.1186/s12893-026-03951-8.

## Introduction

Thoracolithiasis is a rare benign pleural entity characterized by one or more small intrapleural loose bodies (“thoracoliths”), typically calcified and freely mobile within the pleural cavity.

Most reported cases are discovered incidentally during thoracic imaging or at surgery, as patients are usually asymptomatic [1, 2]. In the largest CT-based series to date, Kinoshita et al. reported an incidence of approximately 0.086% among thoracic CT examinations, underscoring the rarity of this entity despite the widespread use of modern imaging [3].

Although thoracolithiasis is clinically benign, failure to recognize it may lead to misinterpretation as malignant or other pleural pathology and precipitate unnecessary invasive diagnostic procedures [4, 5].

In this context, systematic data on non-mobile thoracolithiasis and its clinical correlations are extremely limited. Most available literature consists of single case reports or very small series, and there is a paucity of data on associations with underlying thoracic malignancy, patient demographics, or symptomatology [3, 5, 6] The present study contributes to the limited body of knowledge by presenting a single-center series of 20 patients with non-mobile thoracolithiasis, providing descriptive clinical, radiologic, etiologic, and geographic characteristics. By comparing isolated versus secondary forms and exploring factors such as sex, smoking status, density, symptomatology, and timing of diagnosis, we aim to (1) refine the conceptual framework of thoracolithiasis as a heterogeneous pleural entity, including an examination of its potential correlations with geographic characteristics, and (2) highlight scenarios in which non-mobile thoracolithiasis can closely mimic malignant pleural nodules, leading to potentially avoidable surgical exploration.

## Materials & methods

### Study design and setting

This was a retrospective, observational, descriptive study conducted in the Department of Thoracic Surgery at Ondokuz Mayıs University Faculty of Medicine. Patients diagnosed with thoracolithiasis between January 2010 and December 2024 were reviewed retrospectively.

### Study population

The study population consisted of adult patients who presented to our hospital within the specified period and were found to have imaging findings compatible with thoracolithiasis on thoracic imaging, particularly thoracic computed tomography (CT). Cases diagnosed intraoperatively or confirmed pathologically were also included. Each patient was evaluated only once.

### Inclusion criteria

Patients were eligible for inclusion if they met the following criteria:

- Age ≥ 18 years,
- Diagnosis of thoracolithiasis between January 2010 and December 2024,
- Diagnosis confirmed radiologically (CT, chest X-ray) or surgically,
- Complete availability of clinical records and imaging data,
- While serial imaging for the assessment of lesion mobility was preferred, it was not mandatory for inclusion because this was a retrospective study, and not all patients had interval imaging available.

Both asymptomatic, incidentally detected cases and patients evaluated due to symptoms during the diagnostic process were included.

### Exclusion criteria

Patients were excluded if any of the following applied:

- Age < 18 years,
- Inability to confirm the diagnosis radiologically or surgically,
- Missing or inaccessible imaging (CT, X-ray) or clinical records,
- Cases in which thoracolithiasis remained only a suspected diagnosis and subsequent follow-up revealed an alternative pathology,
- Nodular calcifications clearly attributable to prior invasive pleural procedures (e.g. post–talc pleurodesis),
- Calcified foci arising in the context of secondary malignant pleural disease (e.g. pleural metastases, mesothelioma),
- Patients who were diagnosed at our center but followed exclusively at another institution, with insufficient clinical information available.

### Data collection

Eligible cases were identified through retrospective searches of the hospital information system, picture archiving and communication system (PACS), operative reports, and the pathology database. Keywords used in the search included “thoracolithiasis”, “pleural loose body”, and “mobile pleural nodule”.

For each patient, the following variables were collected:

- Demographic data: age, sex, city of residence, year of presentation, primary reason for presentation.
- Clinical data: presence and type of symptoms, comorbidities, duration of follow-up.
- Radiologic data: type of imaging modality, lesion localization, number of lesions, maximum diameter (mm), density (calcified vs. non-calcified), mobility status, and other concomitant pleural or pulmonary findings.
- Intervention data: clinical follow-up vs. surgical intervention (Video-assisted thoracoscopic surgery [VATS] or thoracotomy), type of surgical procedure, pathology result.
- Diagnostic process: duration of pre-diagnostic follow-up, mode of diagnosis (radiologic vs. surgical).

All data were anonymized and recorded in a Microsoft Excel spreadsheet. Imaging findings relevant to the diagnosis were reviewed independently by more than one investigator, and parameters such as mobility, density, and positional change were assessed by consensus.

### Definitions and radiologic assessment

**Fig. 1:**
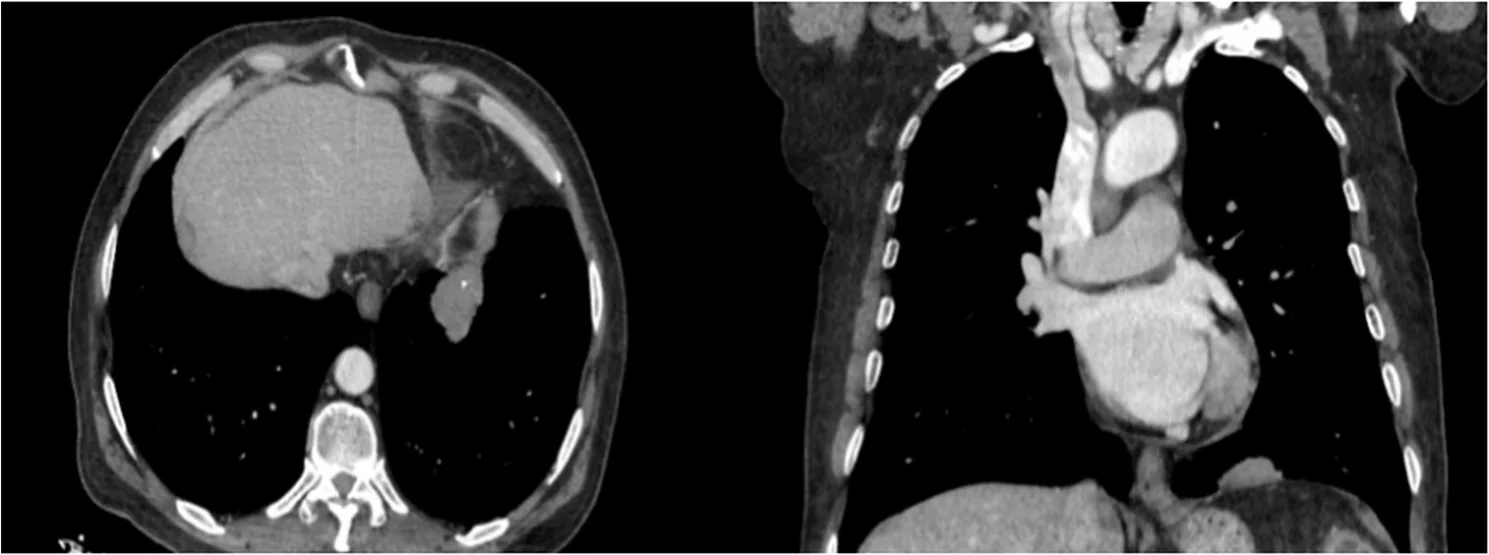
Contrast-enhanced computed tomography showing a well-defined, non-mobile pleural nodule consistent with thoracolithiasis in the left lower hemithorax

**Fig. 2:**
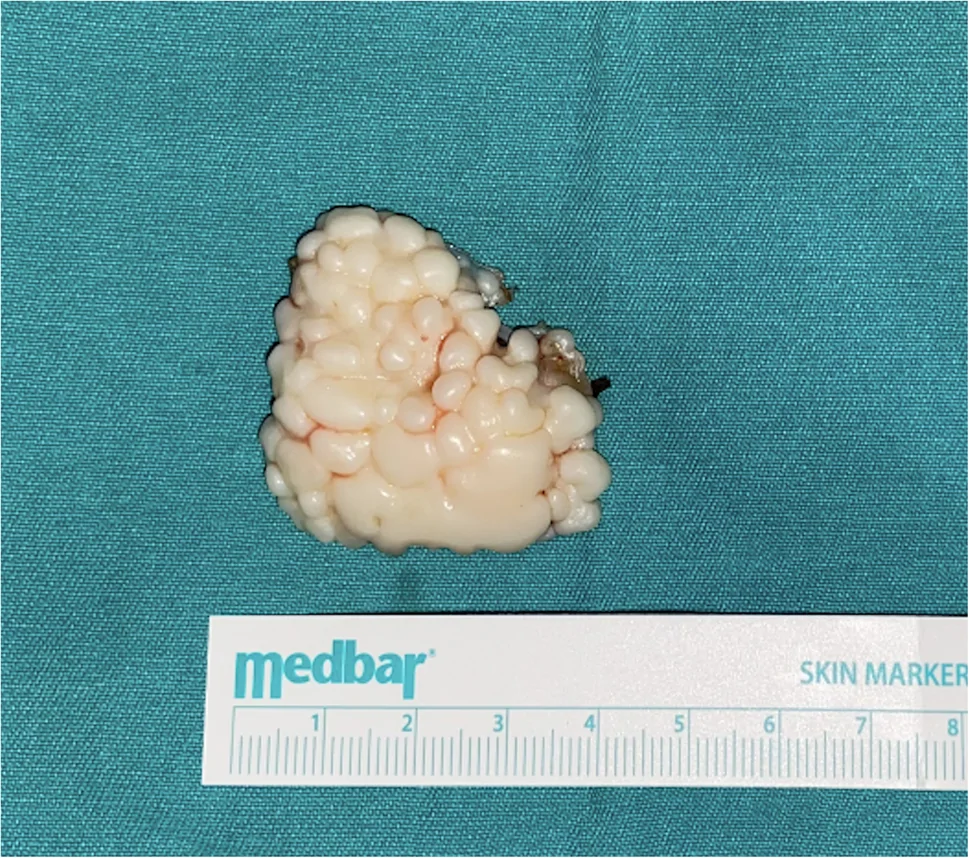
Representative case of isolated, non-malignant thoracolithiasis showing the gross appearance of the excised thoracolith with a smooth, lobulated surface and focal calcifications

The diagnosis of thoracolithiasis was established based on thoracic CT findings showing calcified or non-calcified nodular lesions within the pleural space, confirmed by surgical exploration or follow-up. Figure 1 illustrates a representative chest CT of a non-mobile thoracolithiasis in the left lower hemithorax. The gross morphology of a surgically excised thoracolith from the same patient is shown in Fig. 2, demonstrating a smooth, lobulated surface with focal calcifications.

### Definition of isolated and secondary thoracolithiasis

For analytical purposes, patients were stratified into *isolated* and *secondary* thoracolithiasis based on the presence of concomitant thoracic pathology.

Isolated thoracolithiasis was defined as the presence of thoracolithiasis without any identifiable concomitant thoracic disease on imaging, clinical records, or intraoperative findings.

Secondary thoracolithiasis was defined as thoracolithiasis occurring in patients with coexisting thoracic pathology detected at the time of diagnosis or surgery. These conditions included lung cancer, bullous lung disease, granulomatous disease, pneumothorax, or other clinically significant pleuropulmonary abnormalities. In such cases, thoracoliths were located within the same pleural cavity as the coexisting pathology. 

#### Mobility assessment

Lesion mobility was evaluated retrospectively using available imaging and operative findings. When serial thoracic imaging was available, mobility was assessed by reviewing any positional change of the lesion relative to fixed anatomic landmarks such as the diaphragm, chest wall, fissures, and adjacent pleural surfaces. Lesions were classified as non-mobile when no positional change was observed across available studies. In patients without serial imaging, lack of mobility was inferred from a stable pleural location/fixed appearance on CT and, when applicable, corroborated by intraoperative assessment. Because of the retrospective design, formal mobility assessment was not uniformly available in all patients.

### Histopathologic confirmation

In surgically resected cases, histopathologic examination demonstrated well-circumscribed nodular lesions composed of central necrotic adipose tissue surrounded by dense fibrous tissue, with varying degrees of hyalinization and focal calcification in some cases. No cytological atypia, mitotic activity, spindle-cell proliferation, or features suggestive of mesenchymal neoplasms (e.g., solitary fibrous tumor) were identified. In particular, the absence of cellularity, patternless architecture, or staghorn-like vasculature helped exclude solitary fibrous tumor. In addition, the lesions did not show the broad plaque-like collagenous thickening or characteristic architecture expected in pleural plaques. Dystrophic pleural calcifications and malignant pleural lesions were also excluded based on the combined histopathologic, radiologic, and intraoperative findings. These features, in correlation with the clinical and imaging context, were considered diagnostic of thoracolithiasis.

### Narrative literature review

To contextualize the findings of the present case series, a narrative review of the literature was performed. PubMed and Scopus databases were searched using combinations of the terms “thoracolithiasis”, “pleural loose body”, “intrapleural loose body”, and “calcified pleural nodule”. Relevant English-language publications, including case reports, case series, and radiologic or surgical descriptions of thoracolithiasis, were reviewed. Because the available literature on thoracolithiasis consists predominantly of isolated case reports and small case series, the literature component of this study was designed as a narrative review rather than a formal systematic or scoping review. The retrieved studies were used to summarize reported clinical, radiologic, intraoperative, and histopathologic features and to compare them descriptively with the findings of the present cohort. The final literature search was conducted in January 2026. 

### Ethical approval

This retrospective study was conducted in accordance with the ethical standards of the institutional and national research committees and with the 1964 Declaration of Helsinki and its later amendments. The study protocol was reviewed and approved by the Ondokuz Mayıs University Clinical Research Ethics Committee (Approval No: 2025/239, June 2025). The requirement for informed consent was waived due to the retrospective nature of the study. 

## Statistical analysis

All statistical analyses were performed using IBM SPSS Statistics for Windows, Version 26.0 (IBM Corp., Armonk, NY, USA). Continuous variables were expressed as mean ± standard deviation (SD) or median (interquartile range, IQR), as appropriate. Categorical variables were presented as numbers and percentages [n (%)].

Comparisons between two independent groups (e.g., male vs. female, symptomatic vs. asymptomatic, calcified vs. non-calcified) were performed using the Mann–Whitney U test for continuous variables and the Chi-square or Fisher’s exact test for categorical variables. A two-tailed p-value < 0.05 was considered statistically significant. 

### STROBE compliance

This manuscript was prepared in accordance with the STROBE statement for observational studies. The completed checklist is provided as Supplementary Material. 

## Results

A total of 20 patients with pathologically confirmed thoracolithiasis were included in the study. The detailed demographic, clinical, radiologic, and surgical characteristics of all individual patients are presented in Table 1. The baseline demographic, clinical, radiologic, and surgical characteristics, along with the comparison between isolated and secondary forms, are summarized in Table 2.

**Table 1:** Comprehensive demographic, clinical, radiologic, and surgical characteristics of the study cohort (*N* = 20)

Patient	Age/Sex	Smoker / Asbestos	Comorbidities	Clinical Presentation	Laterality	Max Size (mm) / Density	Coexisting Pathology	Surgical Approach & Intervention	Timing of Diagnosis
1	53 / Female	No / Yes	CAD, Hypothyroidism	Incidental	Left	45 / Non-calcified	None	VATS, Exploration	Intraoperative
2	71 / Female	No / Yes	CAD, Hypothyroidism, RA	Symptomatic (Related - Dyspnea)	Bilateral	16 / Calcified	Fibrotic Nodules	VATS, Exploration	Intraoperative
3	51 / Male	Yes / No	None	Incidental	Left	30 / Non-calcified	Bladder cancer metastasis	VATS, Exploration	Intraoperative
4	70 / Male	Yes / No	DM	Symptomatic (Unrelated - Chest Pain)	Right	60 / Calcified	Lung Cancer	Thoracotomy, Lobectomy	Intraoperative
5	76 / Male	Yes / Yes	Hypothyroidism, HTN	Incidental	Left	30 / Calcified	Lung Cancer	VATS, Exploration	Intraoperative
6	54 / Female	Yes / No	None	Incidental	Left	28 / Calcified	Pneumothorax	Thoracotomy, Wedge	Intraoperative
7	78 / Male	Yes / Yes	CAD	Incidental	Left	12 / Non-calcified	Granulomatous Disease	VATS, Wedge	Preoperative
8	65 / Female	No / No	HTN	Symptomatic (Unrelated - Cough)	Left	6 / Calcified	None	VATS, Exploration	Preoperative
9	69 / Female	No / Yes	CAD, HTN, DM	Symptomatic (Related - Cough)	Right	50 / Non-calcified	None	VATS, Exploration	Intraoperative
10	48 / Male	Yes / No	CAD	Incidental	Right	42 / Non-calcified	None	VATS, Exploration	Intraoperative
11	69 / Male	Yes / No	HTN, DM, BPH	Incidental	Left	37 / Non-calcified	None	VATS, Exploration	Intraoperative
12	75 / Male	Yes / No	None	Symptomatic (Related - Cough)	Right	65 / Calcified	Lung Cancer	VATS, Lobectomy	Preoperative
13	75 / Male	Yes / No	Gastric Cancer	Incidental	Right	20 / Calcified	Lung Cancer	Thoracotomy, Lobectomy	Intraoperative
14	66 / Male	Yes / No	HTN, TB	Symptomatic (Related - Cough)	Right	20 / Non-calcified	Lung Cancer	Thoracotomy, Lobectomy	Preoperative
15	67 / Male	Yes / No	DM, Dysrhythmia, Panic Disorder	Incidental	Right	10 / Non-calcified	Lung Cancer	VATS, Lobectomy	Preoperative
16	64 / Male	Yes / No	COPD, HTN	Incidental	Left	10 / Non-calcified	Bullous Lung Disease	VATS, Wedge	Preoperative
17	74 / Male	Yes / No	None	Incidental	Left	25 / Calcified	Lung Cancer	Thoracotomy, Lobectomy	Preoperative
18	42 / Female	No / No	TB	Symptomatic (Related - Chest Pain)	Right	20 / Non-calcified	None	VATS, Exploration	Intraoperative
19	52 / Male	Yes / No	HTN, Arrhythmia	Symptomatic (Unrelated - Cough)	Right	52 / Non-calcified	Lung Cancer	VATS, Lobectomy	Intraoperative
20	61 / Male	Yes / No	None	Incidental	Right	10 / Non-calcified	Esophageal Cancer	Thoracotomy, Exploration	Intraoperative

None of the lesions demonstrated mobility on available imaging or intraoperative assessment, and no abnormal FDG uptake was observed on PET-CT

*Abbreviations*: *CAD* Coronary artery disease, *DM* Diabetes mellitus, *HTN* Hypertension, *RA* Rheumatoid arthritis, *TB* Tuberculosis, *COPD* Chronic obstructive pulmonary disease, *BPH* Benign prostatic hyperplasia, *VATS* Video-assisted thoracoscopic surgery

**Table 2:** Baseline demographic, clinical, radiologic, and surgical characteristics of the study population and comparison between isolated and secondary thoracolithiasis

Variable	Total Cohort (*N* = 20)	Isolated Thoracolithiasis (*n* = 6)	Secondary Thoracolithiasis (*n* = 14)	*p*-value
Age (years), mean ± SD	64.0 ± 10.5	57.7 ± 11.6	66.7 ± 9.2	0.114
Sex, n (%)				0.037
Male	14 (70.0)	2 (33.3)	12 (85.7)	
Female	6 (30.0)	4 (66.7)	2 (14.3)	
Smoking History, n (%)				0.014
Smoker (active/former)	15 (75.0)	2 (33.3)	13 (92.9)	
Non-smoker	5 (25.0)	4 (66.7)	1 (7.1)	
Lesion Characteristics				
Max. diameter (mm), mean ± SD	29.4 ± 17.8	33.3 ± 16.9	27.7 ± 18.6	0.691
Number of lesions, mean ± SD	1.2 ± 0.8	1.0 ± 0.0	1.4 ± 0.9	0.272
Density, n (%)				0.197
Calcified density	8 (40.0)	1 (16.7)	7 (50.0)	
Non-calcified density	12 (60.0)	5 (83.3)	7 (50.0)	
Clinical Presentation, n (%)				0.598
Symptomatic (thoracolithiasis-related)	5 (25.0)	2 (33.3)	3 (21.4)	
Incidental / Asymptomatic	12 (60.0)	3 (50.0)	9 (64.3)	
Symptoms not attributable to thoracolithiasis	3 (15.0)	1 (16.7)	2 (14.3)	
Surgical Procedure, n (%)				0.025
Diagnostic exploration	10 (50.0)	6 (100.0)	4 (28.6)	
Lobectomy	7 (35.0)	0 (0.0)	7 (50.0)	
Wedge resection	3 (15.0)	0 (0.0)	3 (21.4)	
Timing of Diagnosis, n (%)				0.291
Preoperative (Radiologic)	7 (35.0)	1 (16.7)	6 (42.9)	
Intraoperative (Surgical)	13 (65.0)	5 (83.3)	8 (57.1)	

*SD*  Standard Deviation

*p*-values compare Isolated vs. Secondary groups using the Mann-Whitney U test for continuous variables and Fisher's exact test for categorical variables

### Diagnosis and operative findings

In all included cases, no evidence of lesion mobility was identified on available imaging review and/or intraoperative assessment. Thoracolithiasis was identified preoperatively in 35% of patients and intraoperatively in 65%. Patients with preoperative diagnosis were significantly older (69.9 vs 60.8 years, p = 0.029). Intraoperative detection was more often associated with diagnostic explorations (p = 0.061).

All patients underwent PET (Positron Emission Tomography)-CT as part of the diagnostic work-up. None of the thoracolith lesions demonstrated abnormal fluorodeoxyglucose (FDG) uptake. 

### Demographic features and symptoms

Most patients were residents of Samsun province (12/20, 60%), while the remaining cases were referred from neighboring provinces (Amasya, Corum, Ordu, Tokat) and one patient from Baku, Azerbaijan.

Male and female patients demonstrated similar age, lesion size, and number of thoracoliths; however, significant sex-related differences were observed in smoking history and associated thoracic pathology, as detailed in Table 3.

**Table 3:** Comparison of demographic, clinical, radiologic, and surgical characteristics according to sex

Variable	Male (*n* = 14)	Female (*n* = 6)	*p*-value
Age (years, mean ± SD)	66.1 ± 9.7	59.0 ± 11.8	0.212
Lesion size (mm, mean ± SD)	30.2 ± 18.1	27.5 ± 16.9	0.758
Number of thoracoliths (mean ± SD)	1.3 ± 0.9	1.0 ± 0.7	0.480
Smoking history	14 (100%)	1 (16.7%)	< 0.001
Lung cancer	8 (57.1%)	0 (0%)	0.018
No associated thoracic disease	2 (14.3%)	4 (66.7%)	0.022

*SD* Standard Deviation

Data are presented as mean ± SD or n (%)

Symptomatic and asymptomatic patients showed similar demographic and radiologic characteristics. Minor differences were observed in lesion laterality and detection mode, with symptomatic cases more often right-sided and less frequently incidental (p = 0.031 and p = 0.004, respectively), although these findings had limited clinical significance (Supplementary Table S1). 

### Calcification and coexisting disease

Patients with calcified thoracoliths were significantly older than those with non-calcified lesions (70.0 ± 7.4 vs 60.0 ± 10.7 years, p = 0.023). No significant differences were observed in lesion size, symptom status, smoking history, or associated thoracic pathology between the two groups (Supplementary Table S2).

Patients with coexisting lung cancer were predominantly older male smokers, showing a trend toward higher age compared to those without cancer (69.4 ± 8.2 vs 60.4 ± 10.9 years, p = 0.059). The presence of lung cancer was significantly associated with male sex (p = 0.010) and smoking history (p = 0.027), while other clinical and radiologic features were comparable between groups (Supplementary Table S3). 

### Isolated versus secondary thoracolithiasis

Isolated thoracolithiasis (30% of the cohort) exhibited a distinct clinical profile compared to secondary cases (Table 2). Isolated cases were significantly more common in female non-smokers. Notably, all isolated cases (100%) underwent diagnostic exploration (VATS or thoracotomy), compared to 35.7% in the secondary group (p = 0.025), where resection was often directed at the primary pathology. 

### Asbestos exposure and geography

To explore potential environmental contributors, patients were stratified according to a history of asbestos exposure (5/20, 25%). All asbestos-exposed patients originated from inland provinces (Amasya, Corum, Tokat), whereas most non-exposed patients resided in Samsun (p < 0.001). Coronary artery disease (80.0% vs 6.7%, p = 0.005) and hypothyroidism (60.0% vs 0.0%, p = 0.009) were also more frequent in the asbestos-exposed group; however, given the small number of exposed patients, these findings should be interpreted cautiously. In contrast, asbestos exposure was not clearly associated with lung cancer (20.0% vs 46.7%, p > 0.05) or with differences in symptomatology, lesion size, density, or timing of diagnosis (Supplementary Table S4). 

## Discussion

This single-center 20-patient series adds several important observations to the limited thoracolithiasis literature. First, no lesion in our cohort showed demonstrable mobility on available imaging review or intraoperative assessment, which contrasts with the classical description of thoracolithiasis as a freely mobile intrapleural body [1, 3]. Second, we demonstrate that thoracolithiasis is not a uniform entity but appears to segregate into two clinically distinct patterns: a secondary, male, smoker, cancer-associated type and an isolated, female, non-smoker type. An exploratory geographic pattern was also observed: isolated cases were more often seen in patients residing in non–asbestos-endemic areas, whereas all patients with documented asbestos exposure originated from inland provinces. However, given the small number of asbestos-exposed patients, this observation should be interpreted cautiously.

Third, we show that isolated non-mobile thoracoliths are particularly prone to misdiagnosis as malignant pleural nodules, resulting in a high rate of unnecessary exploratory surgery.

Previous CT-based series and narrative reviews have emphasized three main features of classic thoracolithiasis: rarity, left-sided predominance, and mobility [1, 3, 6]. Kinoshita et al. described 11 cases of calcified intrapleural loose bodies, most located in the left lower hemithorax and all demonstrating mobility on serial imaging [3]. Strzelczyk et al. referred to these lesions as “rolling stones in the pleural space” and proposed that positional change on CT is a key diagnostic criterion [1]. Similarly, Suwatanapongched et al. reported thin-section CT findings in nine patients and confirmed the typical left lower hemithorax distribution, ovoid morphology, and frequent calcification [6]. Gayer also highlighted mobility and laminated internal architecture as important clues to the diagnosis [5].

Although classical thoracolithiasis is defined as a mobile intrapleural loose body, our histopathologically confirmed cases suggest that absence of demonstrable mobility does not necessarily exclude the diagnosis, particularly in lesions with stable pleural attachment, limited interval imaging, or associated pleural adhesions.

In contrast, our series shows a more balanced laterality (right 50%, left 45%) and, importantly, no radiologic or intraoperative mobility in any case. This pattern aligns more closely with recent case reports describing non-mobile thoracolithiasis anchored to pleural fat or presenting as pedunculated-appearing pleural masses, which challenge the classical notion that mobility is obligatory for diagnosis [7–9]. Our data suggest that non-mobile thoracolithiasis may be under-recognized and under-reported, particularly when coexisting with other thoracic pathology.

A growing number of reports document atypical thoracoliths that mimic pleural plaques, metastases, or even primary malignancies [7, 10, 11]. Cha et al. described thoracolithiasis simulating an asbestos-related pleural plaque in an asbestos-exposed patient [7]. Bolca et al. reported a pearl-like thoracolith that closely resembled a pleural or mediastinal tumor [10]. 

Wong et al. recently published a case of thoracolith mimicking pulmonary osteosarcoma metastasis, emphasizing the risk of over-treating this benign entity [11]. Our isolated, non-mobile, mostly non-calcified lesions fit well within this emerging spectrum of “malignancy mimics”. Notably, these atypical presentations clustered among residents of non–asbestos-endemic coastal regions, whereas asbestos-exposed individuals originated exclusively from inland provinces. Although this geographic clustering is intriguing, the very small number of asbestos-exposed patients precludes firm inference. These observations should be regarded as hypothesis-generating and may reflect regional occupational exposure patterns, referral bias, differences in exposure documentation, or chance rather than a true etiologic association.

In our series, all thoracolith lesions were FDG-negative on PET-CT. Although PET imaging is frequently used in the evaluation of pleural nodules and suspected malignancy, published data regarding the PET appearance of thoracolithiasis are extremely limited. Our findings suggest that absence of FDG uptake may represent an additional supportive feature favoring a benign diagnosis. However, FDG-negativity alone should not be considered definitive, particularly in small lesions or in the context of concurrent malignancy.

In our cohort, all six isolated thoracolithiasis cases occurred in non-smoking or low-risk women without coexisting thoracic disease, yet all of these patients (100%) underwent diagnostic exploratory surgery because malignancy could not be confidently excluded preoperatively. This finding parallels prior reports in which non-mobile or atypical thoracoliths were only diagnosed definitively at thoracoscopy or thoracotomy [10, 12, 13]. 

These observations underscore the need to broaden the radiologic and clinical definition of thoracolithiasis beyond the classical mobile, calcified nodule.

In our series, percutaneous needle biopsy was not routinely performed. Given the small size, pleural-based location, and lack of parenchymal extension, transthoracic biopsy was considered to have limited diagnostic yield. In most cases, lesions were either discovered intraoperatively or excised during surgical exploration performed for suspected malignancy or concomitant pathology.

Consistent with previous series, most of our patients were asymptomatic, and symptom presence was not associated with lesion size or calcification [1,2, 14]. Kang et al. and others have reported that symptoms, when present, tend to occur in patients with multiple or larger thoracoliths and often improve after surgical removal [14, 15]. In our series, however, symptomatic cases were defined by a combination of clinical judgment and exclusion of alternative causes, which may partly explain the absence of a clear size–symptom relationship.

More intriguingly, we found that symptoms were associated with right-sided location and a borderline association with prior tuberculosis. These findings suggest that chronic pleural inflammation serves as a common pathophysiological substrate, potentially predisposing patients to both thoracolith formation and mechanical pleural irritation—such as pleuritic pain or cough—by altering local pleural mechanics. Similar associations between thoracolithiasis, prior infection, and pleural adhesions have been noted in individual case reports [15, 16]. 

While causality cannot be established in our small sample, these findings align with one of the major proposed pathogenetic pathways: post-inflammatory organization and detachment of necrotic pleural or pericardial fat [2, 12, 17].

The observed association between calcification and older age in our cohort (70 vs 60 years) supports a time-dependent, degenerative model of thoracolith evolution. Early-stage lesions may be non-calcified, composed primarily of necrotic fat and fibrous tissue, and only gradually develop calcifications over years. This concept is in line with histopathologic descriptions in prior series, where centrally necrotic fat cores showed increasing fibrosis and calcification over time [13, 18]. Our finding that symptom status and lesion size do not differ between calcified and non-calcified groups further suggests that calcification is a marker of chronicity rather than clinical aggressiveness.

Although the term “thoracolithiasis” traditionally implies calcified intrapleural bodies, calcification appears to represent a time-dependent or degenerative feature rather than a mandatory diagnostic criterion. Several prior reports have described non-calcified thoracoliths composed primarily of necrotic fat and fibrous tissue. Despite this limitation in terminology, “thoracolithiasis” remains the established term in radiologic and surgical literature. Rather than proposing a new nomenclature, we suggest that the diagnostic definition be conceptually expanded to include both calcified and non-calcified forms within a mobility and maturation spectrum.

From a radiologic perspective, however, calcification may be a useful clue favoring thoracolithiasis over malignant disease, especially when combined with peripheral pleural location and stability over time [3, 5]. Non-calcified lesions, in contrast, may be more easily mistaken for malignant nodules, particularly when non-mobile and isolated. Indeed, in our cohort, the majority of unnecessary surgical explorations were performed for these non-calcified thoracoliths, as their radiologic appearance frequently mimicked malignancy and raised concern for occult metastatic or primary disease.

The high proportion of patients with coexisting lung cancer in our cohort (40%), all male smokers, indicates that thoracolithiasis may frequently arise in the context of chronic smoking-related and malignant pleural inflammation. Prior CT series and case reports have noted coexisting pulmonary or pleural disease in a substantial subset of thoracolithiasis patients, including emphysema, prior surgery, or malignancy [3, 5, 6]. Our data extend these observations by demonstrating a clear sex- and smoking-linked phenotype:

- Secondary thoracolithiasis in older male smokers with thoracic malignancy or other significant pathology, versus
- Isolated thoracolithiasis in younger or middle-aged non-smoking women without malignancy.

In practical terms, this dichotomy has important implications:

- In high-risk patients (older male smokers with lung cancer), thoracoliths are usually discovered incidentally during oncologic work-up or surgery and rarely influence management.
- In low-risk patients (non-smoking women without cancer), non-mobile thoracoliths are more likely to be interpreted as primary malignant pleural nodules, triggering decision-making cascades that end in diagnostic thoracoscopy or thoracotomy.

Taken together, our findings—supported by recent literature—suggest that the diagnostic concept of thoracolithiasis should be expanded along two axes:

1. Mobility spectrum: Thoracoliths may range from freely mobile to partially adherent to fully non-mobile, particularly when anchored to pleural fat or in the setting of pleural adhesions [7–9].
2. Etiologic spectrum: Thoracolithiasis may represent either an isolated degenerative process in otherwise healthy pleura or a secondary sequel of chronic inflammatory or malignant pleural disease [3, 12, 17].

For radiologists and clinicians, several practical points emerge:

- Non-mobile, pleural-based nodules in low-risk patients—especially non-smoking women without other suspicious findings—should prompt consideration of non-mobile thoracolithiasis in the differential diagnosis, even in the absence of demonstrated mobility.
- Review of prior imaging for subtle change in position, careful assessment of the pleural–parenchymal interface, and attention to fatty or laminated internal structure may provide additional clues [5, 11].
- When thoracolithiasis is strongly suspected and malignancy risk is otherwise low, short-interval imaging follow-up rather than immediate diagnostic thoracoscopy may be reasonable, although this approach requires validation in larger cohorts.

This study has several limitations. It is a single-center, retrospective series with a small sample size (N = 20), which limits statistical power and generalizability. Confidence intervals are wide, and several clinically relevant associations (e.g. tuberculosis history and symptoms, lung cancer and age, lesion size and diagnostic timing) approached but did not reach conventional statistical significance. As our series includes only non-mobile lesions, direct comparison with mobile thoracolithiasis was not possible. Finally, referral and selection bias are likely, as all patients ultimately underwent surgery, potentially over-representing diagnostically challenging or symptomatic cases. 

## Conclusion

In this 20-patient series, thoracolithiasis presented exclusively as non-mobile pleural nodules, challenging the traditional view that mobility is a mandatory diagnostic feature. Our data support the existence of two clinically distinct patterns:

1. A secondary type, occurring predominantly in older male smokers with lung cancer or other significant thoracic pathology; and
2. An isolated type, seen mainly in non-smoking women without additional thoracic disease, in whom non-mobile thoracoliths frequently mimicked malignant pleural nodules and led to unnecessary diagnostic exploration.

Calcification was associated with older age, consistent with a time-dependent degenerative process, whereas symptomatology appeared more closely related to side (right hemithorax) and prior tuberculosis than to lesion size or density. Recognition that thoracolithiasis may be non-mobile, non-calcified, and isolated—particularly in low-risk patients—is crucial to avoid misdiagnosis and potentially prevent avoidable invasive procedures.

Future multicenter studies with larger cohorts, including both mobile and non-mobile lesions, are needed to refine diagnostic criteria and to develop evidence-based management strategies for this rare but clinically relevant pleural entity. 

## Supplementary Information

Supplementary Material 1.

Supplementary Material 2.

Supplementary Material 3.

Supplementary Material 4.

Supplementary Material 5.

## Data Availability

The datasets generated and/or analyzed during the current study are available from the corresponding author on reasonable request.
