# Supplementary material for: Thoracolithiasis: a rare and frequently underrecognized entity – a retrospective single-center study and narrative literature review
Source: BMC Surg. 2026 Jun 15;26:563. doi: 10.1186/s12893-026-03951-8 (PMC13495144; doi:10.1186/s12893-026-03951-8)
Supplement: Supplementary file 1 [file 12893_2026_3951_MOESM1_ESM.docx]

The STROBE reporting checklist

For checking that observational epidemiology research articles can be understood and used by everyone

 Note

If you have not used a reporting guideline before, read about [how and why to use them](https:/resources.equator-network.org/about/reporting-guidelines.html) and check whether STROBE is the [most applicable reporting guideline](https:/resources.equator-network.org/reporting-guidelines/strobe/index.html?#applicability) for your work.

Reporting guidelines are most useful when used early in research. When writing a manuscript or application, consider using the [Full Guidance](https:/resources.equator-network.org/reporting-guidelines/strobe/index.html) where you’ll see explanations and examples for each item.

After writing, demonstrate adherence by completing this checklist:

1. Specify where each item is described (see Note 1).
2. Cite this checklist (See Note 2).
3. Include your completed checklist as a supplement when submitting to a journal so that future readers can use it to find information.

|  | Item Description | Location (or reason for not reporting) |
| --- | --- | --- |
| **Title and abstract** |  |  |
| [1a. Indicate the study’s design](https:/resources.equator-network.org/reporting-guidelines/strobe/items/title-abstract-indicate-study-design.html?utm_source=strobe&utm_medium=checklist&utm_campaign=1_1) | Indicate the study’s design with a commonly used term in the title or the abstract. | **Location:**   - Title - Abstract, Methods (first sentence) - Materials & Methods – “Study Design and Setting”, paragraph 1   **Statement:** The study design (“retrospective, observational study”) is clearly stated in the Abstract (Methods section) and explicitly described in the Materials & Methods section under “Study Design and Setting.” |
| [1b. Abstract](https:/resources.equator-network.org/reporting-guidelines/strobe/items/abstract.html?utm_source=strobe&utm_medium=checklist&utm_campaign=1_1) | Provide in the abstract an informative and balanced summary of what was done and what was found. | **Location:**  Abstract (structured format: Objectives, Materials and Methods, Results, Conclusion)  **Statement:**  The abstract provides a structured and balanced summary of the study objectives, design, setting, eligibility criteria, statistical methods, key numerical results (including sample size and significant p-values), and principal conclusions. |
| **Introduction** |  |  |
| [2. Background / rationale](https:/resources.equator-network.org/reporting-guidelines/strobe/items/background-rationale.html?utm_source=strobe&utm_medium=checklist&utm_campaign=1_1) | Explain the scientific background and rationale for the investigation being reported. | **Location:**  Introduction, paragraphs 1–3  **Statement:**  The Introduction explains the scientific background of thoracolithiasis, summarizes existing literature (including CT-based series and case reports), and clearly identifies knowledge gaps, particularly regarding non-mobile thoracolithiasis and its potential to mimic malignant pleural disease. The clinical importance of avoiding unnecessary invasive procedures is explicitly stated. |
| [3. Objectives](https:/resources.equator-network.org/reporting-guidelines/strobe/items/objectives.html?utm_source=strobe&utm_medium=checklist&utm_campaign=1_1) | State specific objectives, including any prespecified hypotheses. | **Location:**  Introduction, final paragraph  **Statement:**  The study objectives are clearly stated, including:   - Characterization of clinical, radiologic, etiologic, and geographic features of non-mobile thoracolithiasis - Comparison of isolated versus secondary forms - Identification of scenarios in which thoracolithiasis mimics malignant pleural nodules   Although no formal hypothesis-testing model was prespecified, the study aimed to explore phenotypic and etiologic patterns within this rare entity. |
| **Methods** |  |  |
| [4. Study design](https:/resources.equator-network.org/reporting-guidelines/strobe/items/study-design.html?utm_source=strobe&utm_medium=checklist&utm_campaign=1_1) | Present key elements of study design early in the paper. | **Location:**  Materials & Methods – “Study Design and Setting”, paragraph 1  **Statement:**  The study design is explicitly described as a retrospective, observational, descriptive study conducted at a single tertiary thoracic surgery center between January 2010 and December 2024. |
| [5. Setting](https:/resources.equator-network.org/reporting-guidelines/strobe/items/setting.html?utm_source=strobe&utm_medium=checklist&utm_campaign=1_1) | Describe the setting, locations, and relevant dates, including periods of recruitment, exposure, follow-up, and data collection. | **Location:**   - Materials & Methods – “Study Design and Setting” - Results – first paragraph   **Statement:**  The setting (Department of Thoracic Surgery, Ondokuz Mayıs University Faculty of Medicine) and the study period (January 2010–December 2024) are clearly specified. The geographic distribution of patients is detailed in the Results section (Demographic Features and Symptoms). |
| [6a. Eligibility criteria](https:/resources.equator-network.org/reporting-guidelines/strobe/items/eligibility-criteria.html?utm_source=strobe&utm_medium=checklist&utm_campaign=1_1) | **Cohort study:** Give the eligibility criteria, and the sources and methods of selection of participants. Describe methods of follow-up. **Case-control study:** Give the eligibility criteria, and the sources and methods of case ascertainment and control selection. Give the rationale for the choice of cases and controls. **Cross-sectional study:** Give the eligibility criteria, and the sources and methods of selection of participants. | **Location:**   - Materials & Methods – “Study Population” - “Inclusion Criteria” - “Exclusion Criteria”   **Statement:** Eligibility criteria are explicitly defined, including:   - Age ≥ 18 years - Radiologic and/or surgical confirmation - Complete imaging and clinical data   Exclusion criteria are clearly listed, including:   - Alternative diagnosis on follow-up - Post-procedural pleural calcifications - Secondary malignant pleural calcifications - Incomplete data   Case identification was performed through hospital information systems, PACS, operative reports, and pathology databases using predefined keywords. |
| [6b. Matching criteria](https:/resources.equator-network.org/reporting-guidelines/strobe/items/matching-criteria.html?utm_source=strobe&utm_medium=checklist&utm_campaign=1_1) | **Cohort study:** For matched studies, give matching criteria and number of exposed and unexposed. **Case-control study:** For matched studies, give matching criteria and the number of controls per case. | Not applicable. No matching or comparison cohort was used. |
| [7. Variables](https:/resources.equator-network.org/reporting-guidelines/strobe/items/variables.html?utm_source=strobe&utm_medium=checklist&utm_campaign=1_1) | Clearly define all outcomes, exposures, predictors, potential confounders, and effect modifiers. Give diagnostic criteria, if applicable. | **Location:**   - Materials & Methods – “Data Collection” - “Definitions and Radiologic Assessment”   **Statement:** The study clearly defines:  **Exposure variables:**   - Sex - Smoking history - Asbestos exposure - Presence of coexisting lung cancer - Tuberculosis history   **Outcome variables:**   - Symptom status - Need for surgical exploration - Mode of diagnosis - Calcification status - Lesion mobility   Radiologic definitions (mobility, density, localization) are described in the “Definitions and Radiologic Assessment” subsection. |
| [8. Data sources / measurement](https:/resources.equator-network.org/reporting-guidelines/strobe/items/data-sources-measurement.html?utm_source=strobe&utm_medium=checklist&utm_campaign=1_1) | For each variable of interest give sources of data and details of methods of assessment (measurement). Describe comparability of assessment methods if there is more than one group. | **Location:**   - Materials & Methods – “Data Collection” - “Definitions and Radiologic Assessment” - “Histopathologic Confirmation”   **Statement:** Data were obtained from:   - Hospital information system - PACS - Operative reports - Pathology database   Imaging parameters (mobility, density, lesion size) were evaluated independently by more than one investigator and assessed by consensus. Histopathologic criteria for thoracolithiasis are explicitly described. |
| [9. Bias](https:/resources.equator-network.org/reporting-guidelines/strobe/items/bias.html?utm_source=strobe&utm_medium=checklist&utm_campaign=1_1) | Describe any efforts to address potential sources of bias. | **Location:**   - Discussion – “Limitations” section   **Statement:** Potential sources of bias are acknowledged, including:   - Single-center design - Small sample size - Selection bias (all patients underwent surgery) - Referral bias - Lack of mobile thoracolith comparison group   The retrospective nature of the study is explicitly discussed as a limitation. |
| [10. Study size](https:/resources.equator-network.org/reporting-guidelines/strobe/items/study-size.html?utm_source=strobe&utm_medium=checklist&utm_campaign=1_1) | Explain how the study size was arrived at. | **Location:**   - Materials & Methods – Study Population - Results – first paragraph   **Statement:** The study included all eligible patients diagnosed with thoracolithiasis during the study period (January 2010–December 2024). No a priori sample size calculation was performed due to the rarity of the condition. |
| [11. Quantitative variables](https:/resources.equator-network.org/reporting-guidelines/strobe/items/quantitative-variables.html?utm_source=strobe&utm_medium=checklist&utm_campaign=1_1) | Explain how quantitative variables were handled in the analyses. If applicable, describe which groupings were chosen, and why. | **Location:**   - Statistical Analysis - Results – relevant subsections   **Statement:** Continuous variables (e.g., age, lesion size) were treated as continuous data and expressed as mean ± SD. Categorical variables were analyzed as proportions. Calcification status was treated as a binary variable (calcified vs non-calcified). Etiologic classification was predefined as isolated vs secondary thoracolithiasis. |
| [12a. Statistical methods](https:/resources.equator-network.org/reporting-guidelines/strobe/items/statistical-methods-description.html?utm_source=strobe&utm_medium=checklist&utm_campaign=1_1) | Describe all statistical methods, including those used to control for confounding. | **Location:**   - Statistical Analysis section   **Statement:** Statistical analysis was performed using IBM SPSS Statistics Version 26. Comparisons between two independent groups were performed using:   - Mann–Whitney U test (continuous variables) - Chi-square or Fisher’s exact test (categorical variables)   A two-tailed p-value < 0.05 was considered statistically significant. |
| [12b. Statistical methods – subgroups and interactions](https:/resources.equator-network.org/reporting-guidelines/strobe/items/statistical-methods-subgroups-interactions.html?utm_source=strobe&utm_medium=checklist&utm_campaign=1_1) | Describe any methods used to examine subgroups and interactions. | **Location:**   - Results – Calcification and Coexisting Disease - Results – Isolated vs Secondary Thoracolithiasis - Results – Asbestos Exposure   **Statement:** Predefined subgroup analyses were performed based on:   - Sex - Calcification - Lung cancer status - Isolated vs secondary thoracolithiasis - Asbestos exposure |
| [12c. Statistical methods – missing data](https:/resources.equator-network.org/reporting-guidelines/strobe/items/statistical-methods-missing-data.html?utm_source=strobe&utm_medium=checklist&utm_campaign=1_1) | Explain how missing data were addressed. | **Location:**   - Materials & Methods – Inclusion Criteria   **Statement:** Cases with incomplete imaging or clinical data were excluded from analysis. No imputation was performed. |
| [12di. Statistical methods – loss to follow-up](https:/resources.equator-network.org/reporting-guidelines/strobe/items/statistical-methods-loss-to-follow-up.html?utm_source=strobe&utm_medium=checklist&utm_campaign=1_1) | **Cohort study:** If applicable, describe how loss to follow-up was addressed. | Not applicable. This retrospective study did not include prospective follow-up or longitudinal outcome assessment. |
| [12dii. Statistical methods – matching cases and controls](https:/resources.equator-network.org/reporting-guidelines/strobe/items/statistical-methods-matching-cases-controls.html?utm_source=strobe&utm_medium=checklist&utm_campaign=1_1) | **Case-control study:** If applicable, explain how matching of cases and controls was addressed. | Not applicable. This was not a matched case-control study. |
| [12diii. Statistical methods – sampling strategy](https:/resources.equator-network.org/reporting-guidelines/strobe/items/statistical-methods-analytical-methods-sampling-strategy.html?utm_source=strobe&utm_medium=checklist&utm_campaign=1_1) | **Cross-sectional study:** If applicable, describe analytical methods taking account of sampling strategy. | Not applicable. This was not a cross-sectional study with sampling strategy. |
| [12e. Statistical methods – sensitivity analyses](https:/resources.equator-network.org/reporting-guidelines/strobe/items/statistical-methods-sensitivity-analyses.html?utm_source=strobe&utm_medium=checklist&utm_campaign=1_1) | Describe any sensitivity analyses. | No sensitivity analyses were performed due to the small sample size and descriptive nature of the study. |
| **Results** |  |  |
| [13a. Participant numbers](https:/resources.equator-network.org/reporting-guidelines/strobe/items/participants-numbers.html?utm_source=strobe&utm_medium=checklist&utm_campaign=1_1) | Report the numbers of individuals at each stage of the study—e.g., numbers potentially eligible, examined for eligibility, confirmed eligible, included in the study, completing follow-up, and analysed; Consider use of a flow diagram. | **Location:**   - Results – first paragraph   **Statement:** A total of 20 adult patients with pathologically confirmed thoracolithiasis diagnosed between January 2010 and December 2024 were included in the final analysis.  All included patients met predefined inclusion criteria and had complete clinical and imaging data. |
| [13b. Participants – non-participation](https:/resources.equator-network.org/reporting-guidelines/strobe/items/participants-non-participation.html?utm_source=strobe&utm_medium=checklist&utm_campaign=1_1) | Give reasons for non-participation at each stage. | **Location:**   - Materials & Methods – Exclusion Criteria   **Statement:** Patients were excluded if:   - Diagnosis could not be confirmed radiologically or surgically - Imaging or clinical data were incomplete - Follow-up revealed alternative pathology - Calcifications were attributable to prior pleural procedures - Secondary malignant pleural calcifications were present   Exact numbers of excluded patients were not available due to retrospective database-based identification. |
| [13c. Participants – flow diagram](https:/resources.equator-network.org/reporting-guidelines/strobe/items/participants-flow-diagram.html?utm_source=strobe&utm_medium=checklist&utm_campaign=1_1) | Consider use of a flow diagram. | Not provided. Given the small sample size (N = 20) and retrospective identification, a formal flow diagram was not constructed. |
| [14a. Descriptive data – participant characteristics](https:/resources.equator-network.org/reporting-guidelines/strobe/items/descriptive-data-participant-characteristics.html?utm_source=strobe&utm_medium=checklist&utm_campaign=1_1) | Give characteristics of study participants (e.g., demographic, clinical, social) and information on exposures and potential confounders. Present the information in a table. | **Location:**   - Results – first paragraph - Table 1 - Table 2   **Statement:** Demographic, clinical, radiologic, and surgical characteristics are summarized in Table 1.  Key characteristics:   - Mean age: 64.0 ± 10.5 years - 70% male - 75% smoking history - 40% coexisting lung cancer - 30% isolated thoracolithiasis - 25% asbestos exposure   Comparisons by sex are presented in Table 2. |
| [14b. Descriptive data – missing data](https:/resources.equator-network.org/reporting-guidelines/strobe/items/descriptive-data-missing-data.html?utm_source=strobe&utm_medium=checklist&utm_campaign=1_1) | Indicate the number of participants with missing data for each variable of interest. | **Location:**   - Materials & Methods – Inclusion Criteria   **Statement:** Only patients with complete clinical and imaging data were included. No variable included in the final analysis had missing data. |
| [14c. Descriptive data – follow-up time](https:/resources.equator-network.org/reporting-guidelines/strobe/items/descriptive-data-follow-up-time.html?utm_source=strobe&utm_medium=checklist&utm_campaign=1_1) | **Cohort study:** Summarise follow-up time—e.g., average and total amount. | **Location:**   - Materials & Methods – Data Collection - Results (diagnostic timing discussion)   **Statement:** Duration of follow-up was recorded where available; however, formal time-to-event analysis was not performed due to the descriptive nature of the study and small sample size. |
| [15. Outcome data](https:/resources.equator-network.org/reporting-guidelines/strobe/items/outcome-data.html?utm_source=strobe&utm_medium=checklist&utm_campaign=1_1) | **Cohort study:** Report numbers of outcome events or summary measures over time. **Case-control study:** Report numbers in each exposure category, or summary measures of exposure. **Cross-sectional study:** Report numbers of outcome events or summary measures. | **Location:**   - Results – Diagnosis and Operative Findings - Results – Calcification and Coexisting Disease - Results – Isolated vs Secondary Thoracolithiasis   **Statement:** Primary descriptive outcomes included:   - Mobility status (100% non-mobile) - Calcification status (60% non-calcified) - Mode of diagnosis (35% preoperative; 65% intraoperative) - Surgical exploration rate (100% in isolated cases vs 35.7% in secondary cases, p = .0253)   All thoracolith lesions were FDG-negative on PET-CT. |
| [16a. Main results](https:/resources.equator-network.org/reporting-guidelines/strobe/items/main-results.html?utm_source=strobe&utm_medium=checklist&utm_campaign=1_1) | Give unadjusted estimates and, if applicable, confounder-adjusted estimates and their precision (e.g., 95% confidence intervals). Make clear which confounders were adjusted for and why they were included. | **Location:**   - Results – relevant subsections   **Statement:** Associations were reported with corresponding p-values:   - Calcified lesions associated with older age (70.0 vs 60.0 years, p = .023) - Lung cancer associated with male sex (p = .010) - Lung cancer associated with smoking history (p = .027) - Isolated thoracolithiasis associated with diagnostic exploration (p = .0253) - Geographic clustering with asbestos exposure (p = .0004)   Effect estimates were unadjusted due to small sample size. |
| [16b. Main results – category boundaries](https:/resources.equator-network.org/reporting-guidelines/strobe/items/main-results-category-boundaries.html?utm_source=strobe&utm_medium=checklist&utm_campaign=1_1) | Report category boundaries when continuous variables were categorised. | **Location:**   - Materials & Methods – Definitions and Radiologic Assessment - Statistical Analysis   **Statement:** Predefined binary classifications included:   - Calcified vs non-calcified - Isolated vs secondary thoracolithiasis - Symptomatic vs asymptomatic - Asbestos exposure (yes/no) - Smoking history (yes/no)   No post hoc categorization was introduced. |
| [16c. Main results – risk](https:/resources.equator-network.org/reporting-guidelines/strobe/items/main-results-risk.html?utm_source=strobe&utm_medium=checklist&utm_campaign=1_1) | If relevant, consider translating estimates of relative risk into absolute risk for a meaningful time period. | Not applicable. No risk ratios or regression models were calculated due to small sample size. |
| [17. Other analyses](https:/resources.equator-network.org/reporting-guidelines/strobe/items/other-analyses.html?utm_source=strobe&utm_medium=checklist&utm_campaign=1_1) | Report other analyses done—e.g., analyses of subgroups and interactions, and sensitivity analyses. | **Location:**   - Results – Isolated vs Secondary Thoracolithiasis - Results – Asbestos Exposure - Results – Calcification and Coexisting Disease   **Statement:** Predefined subgroup analyses were performed according to:   - Sex - Smoking status - Calcification - Lung cancer presence - Etiologic classification - Asbestos exposure   No multivariable or sensitivity analyses were conducted due to limited sample size. |
| **Discussion** |  |  |
| [18. Key results](https:/resources.equator-network.org/reporting-guidelines/strobe/items/key-results.html?utm_source=strobe&utm_medium=checklist&utm_campaign=1_1) | Summarise key results with reference to study objectives. | **Location:**   - Discussion – first paragraph - Conclusion   **Statement:** The Discussion begins with a clear summary of the principal findings in relation to the predefined objectives.  Specifically:   - All lesions were non-mobile, challenging the classical definition of thoracolithiasis. - Two distinct clinical–etiologic phenotypes were identified (secondary vs isolated). - Non-mobile, non-calcified thoracoliths in low-risk patients frequently mimicked malignant pleural nodules. - All lesions were FDG-negative on PET-CT. - Calcification was associated with older age, supporting a degenerative maturation model.   The Conclusion reiterates these findings and aligns them with the study objectives. |
| [19. Limitations](https:/resources.equator-network.org/reporting-guidelines/strobe/items/limitations.html?utm_source=strobe&utm_medium=checklist&utm_campaign=1_1) | Discuss limitations of the study, taking into account sources of potential bias or imprecision. Discuss both direction and magnitude of any potential bias. | **Location:**   - Discussion – “Limitations” section   **Statement:** The limitations are explicitly acknowledged, including:   - Single-center design - Retrospective observational methodology - Small sample size (N = 20) - Limited statistical power - Absence of mobile thoracolith cases - Referral and selection bias (all patients underwent surgery) - Lack of multivariable analysis - Wide confidence intervals - Associations that approached but did not reach statistical significance   The potential direction and magnitude of bias are discussed. |
| [20. Interpretation](https:/resources.equator-network.org/reporting-guidelines/strobe/items/interpretation.html?utm_source=strobe&utm_medium=checklist&utm_campaign=1_1) | Give a cautious overall interpretation considering objectives, limitations, multiplicity of analyses, results from similar studies, and other relevant evidence. | **Location:**   - Discussion – full section - Conclusion   **Statement:** Interpretation is cautious and proportional to the study design.  The manuscript:   - Avoids causal claims - Emphasizes hypothesis-generating nature - Compares findings with prior CT-based series and case reports - Discusses alternative explanations (degenerative theory, inflammatory hypothesis) - Notes scarcity of PET literature - Explicitly states need for multicenter validation   No overgeneralization is made. |
| [21. Generalisability](https:/resources.equator-network.org/reporting-guidelines/strobe/items/generalisability.html?utm_source=strobe&utm_medium=checklist&utm_campaign=1_1) | Discuss the generalisability (external validity) of the study results. | **Location:**   - Discussion – final paragraphs - Limitations section   **Statement:** The manuscript acknowledges limited generalizability due to:   - Small cohort - Single tertiary center - Inclusion of only non-mobile lesions - Surgical selection bias   It suggests that findings may primarily apply to:   - Non-mobile thoracolithiasis - Tertiary referral populations - Regions with similar asbestos exposure patterns   Future multicenter studies are recommended. |
| **Other information** |  |  |
| [22. Funding](https:/resources.equator-network.org/reporting-guidelines/strobe/items/funding.html?utm_source=strobe&utm_medium=checklist&utm_campaign=1_1) | Give the source of funding and the role of the funders for the present study and, if applicable, for the original study on which the present article is based. | **Location:** Declarations – Funding section  **Statement in manuscript:** “The authors received no specific funding for this work. No funding body had any role in the design of the study; collection, analysis, and interpretation of data; or in writing the manuscript.” |

## 1 How to specify where content is

Tell the reader where they can find information. E.g.,

- Results; paragraph 2
- Methods, Participants; paragraphs 1 & 2.
- Table 3
- Supplement B, para. 4

If you have chosen not to describe an item, explain why. You can do this in the checklist, or as a note below it.

You can describe items in the article body, or in tables, figures, or supplementary materials, and should prioritize items you feel are most important to your intended audience. The order of items in your manuscript does not need to match the order of items in this checklist. You can decide how best to structure your work.

## 2 How to cite

Describe how you used STROBE at the end of your Methods section, referencing the resources you used e.g.,

‘We used the STROBE reporting guideline(1) to draft this manuscript, and the STROBE reporting checklist(2) when editing, included in supplement A’

If you use a reporting checklist, remember to include it as a supplement when publishing so that readers can easily find information and see how you have interpreted the guidance.

1.	Elm E von, Altman DG, Egger M, Pocock SJ, Gøtzsche PC, Vandenbroucke JP, et al. The strengthening the reporting of observational studies in epidemiology (STROBE) statement: Guidelines for reporting observational studies. Annals of Internal Medicine [Internet]. 2007 Oct;147(8):573–7. Available from: <https://www.acpjournals.org/doi/10.7326/0003-4819-147-8-200710160-00010>

2.	Elm E von, Altman DG, Egger M, Pocock SJ, Gøtzsche PC, Vandenbroucke JP, et al. The STROBE reporting checklist. In: Harwood J, Albury C, Beyer J de, Schlüssel M, Collins G, editors. The EQUATOR network reporting guideline platform [Internet]. The UK EQUATOR Centre; 2025. Available from: [https:/resources.equator-network.org/reporting-guidelines/strobe/strobe-checklist.docx](https://https:/resources.equator-network.org/reporting-guidelines/strobe/strobe-checklist.docx)
