# Supplementary material for: Thoracolithiasis: a rare and frequently underrecognized entity – a retrospective single-center study and narrative literature review
Source: BMC Surg. 2026 Jun 15;26:563. doi: 10.1186/s12893-026-03951-8 (PMC13495144; doi:10.1186/s12893-026-03951-8)
Supplement: Supplementary file 2 [file 12893_2026_3951_MOESM2_ESM.docx]

**Supplementary Table S1.** Comparison of demographic, clinical, radiologic, and surgical characteristics according to symptom status

| **Variable** | **Symptomatic (n = 5)** | **Asymptomatic (n = 15)** | **p-value** |
| --- | --- | --- | --- |
| **Age (years)**, mean ± SD | 64.6 ± 13.0 | 63.8 ± 10.1 | 0.905 |
| **Sex (Female)**, n (%) | 3 (60.0) | 3 (20.0) | 0.127 |
| **Smoking History**, n (%) | 2 (40.0) | 13 (86.7) | 0.065 |
| **Lesion Size (mm)**, mean ± SD | 34.2 ± 22.0 | 27.8 ± 16.8 | 0.575 |
| **Density (Calcified)**, n (%) | 2 (40.0) | 6 (40.0) | 1.000 |
| **Laterality**, n (%) |  |  | 0.031 |
| Right | 4 (80.0) | 6 (40.0) |  |
| Left | 0 (0.0) | 9 (60.0) |  |
| Bilateral | 1 (20.0) | 0 (0.0) |  |
| **Coexisting Lung Cancer**, n (%) | 2 (40.0) | 6 (40.0) | 1.000 |
| **Tuberculosis History**, n (%) | 2 (40.0) | 0 (0.0) | 0.053 |
| **Incidental Diagnosis**, n (%) | 0 (0.0) | 12 (80.0) | 0.004 |
| **Surgical Procedure**, n (%) |  |  | 0.749 |
| Exploration | 3 (60.0) | 7 (46.7) |  |
| Lobectomy | 2 (40.0) | 5 (33.3) |  |
| Wedge Resection | 0 (0.0) | 3 (20.0) |  |

**Note:** SD = Standard Deviation. *p*-values are derived from the Mann-Whitney U test for continuous variables and Fisher's exact test for categorical variables.
For subgroup analysis, patients were dichotomized as those with thoracolithiasis-related symptoms (n=5) and those without such symptoms (including incidental/asymptomatic patients and those with symptoms not attributable to thoracolithiasis) (n=15).
