# Supplementary material for: Thoracolithiasis: a rare and frequently underrecognized entity – a retrospective single-center study and narrative literature review
Source: BMC Surg. 2026 Jun 15;26:563. doi: 10.1186/s12893-026-03951-8 (PMC13495144; doi:10.1186/s12893-026-03951-8)
Supplement: Supplementary file 3 [file 12893_2026_3951_MOESM3_ESM.docx]

**Supplementary Table S2.** Comparison of demographic, clinical, radiologic, and surgical characteristics according to lesion density

| **Variable** | **Calcified (n = 8)** | **Non-calcified (n = 12)** | **p-value** |
| --- | --- | --- | --- |
| **Age (years)**, mean ± SD | 70.0 ± 7.4 | 60.0 ± 10.7 | 0.023 |
| **Sex (Female)**, n (%) | 3 (37.5) | 3 (25.0) | 0.642 |
| **Smoking History**, n (%) | 6 (75.0) | 9 (75.0) | 1.000 |
| **Lesion Size (mm)**, mean ± SD | 31.2 ± 20.7 | 28.2 ± 16.5 | 0.740 |
| **Symptomatic Presentation**, n (%) | 2 (25.0) | 3 (25.0) | 1.000 |
| **Coexisting Lung Cancer**, n (%) | 5 (62.5) | 3 (25.0) | 0.184 |
| **Surgical Procedure**, n (%) |  |  | 0.161 |
| Exploration | 3 (37.5) | 7 (58.3) |  |
| Lobectomy | 4 (50.0) | 3 (25.0) |  |
| Wedge Resection | 1 (12.5) | 2 (16.7) |  |
| **Timing of Diagnosis**, n (%) |  |  | 1.000 |
| Preoperative | 3 (37.5) | 4 (33.3) |  |
| Intraoperative | 5 (62.5) | 8 (66.7) |  |

**Note:** SD = Standard Deviation. *p*-values are derived from the Mann-Whitney U test for continuous variables and Fisher's exact test for categorical variables.
