# Supplementary material for: Thoracolithiasis: a rare and frequently underrecognized entity – a retrospective single-center study and narrative literature review
Source: BMC Surg. 2026 Jun 15;26:563. doi: 10.1186/s12893-026-03951-8 (PMC13495144; doi:10.1186/s12893-026-03951-8)
Supplement: Supplementary file 4 [file 12893_2026_3951_MOESM4_ESM.docx]

**Supplementary Table S3.** Comparison of demographic, clinical, radiologic, and surgical characteristics according to the presence of coexisting lung cancer

| **Variable** | **Lung Cancer (n = 8)** | **No Lung Cancer (n = 12)** | **p-value** |
| --- | --- | --- | --- |
| **Age (years)**, mean ± SD | 69.4 ± 8.0 | 60.4 ± 10.8 | 0.059 |
| **Sex (Male)**, n (%) | 8 (100.0) | 6 (50.0) | 0.042 |
| **Smoking History**, n (%) | 8 (100.0) | 7 (58.3) | 0.055 |
| **Lesion Size (mm)**, mean ± SD | 35.2 ± 20.7 | 25.5 ± 15.3 | 0.277 |
| **Density (Calcified)**, n (%) | 5 (62.5) | 3 (25.0) | 0.184 |
| **Symptomatic Presentation**, n (%) | 2 (25.0) | 3 (25.0) | 1.000 |
| **Coronary Artery Disease**, n (%) | 0 (0.0) | 5 (41.7) | 0.055 |
| **Surgical Procedure**, n (%) |  |  | < 0.001 |
| Exploration | 1 (12.5) | 9 (75.0) |  |
| Lobectomy | 7 (87.5) | 0 (0.0) |  |
| Wedge Resection | 0 (0.0) | 3 (25.0) |  |

**Note:** SD = Standard Deviation. *p*-values are derived from the Mann-Whitney U test for continuous variables and Fisher's exact test for categorical variables.
