# Supplementary material for: Thoracolithiasis: a rare and frequently underrecognized entity – a retrospective single-center study and narrative literature review
Source: BMC Surg. 2026 Jun 15;26:563. doi: 10.1186/s12893-026-03951-8 (PMC13495144; doi:10.1186/s12893-026-03951-8)
Supplement: Supplementary file 5 [file 12893_2026_3951_MOESM5_ESM.docx]

**Supplementary Table S4.** Comparison of demographic, clinical, radiologic, and surgical characteristics according to asbestos exposure

| **Variable** | **Asbestos Exposed (n = 5)** | **Non-exposed (n = 15)** | **p-value** |
| --- | --- | --- | --- |
| **Age (years)**, mean ± SD | 69.4 ± 9.9 | 62.2 ± 10.5 | 0.199 |
| **Sex (Female)**, n (%) | 3 (60.0) | 3 (20.0) | 0.263 |
| **Smoking History**, n (%) | 2 (40.0) | 13 (86.7) | 0.065 |
| **Lesion Size (mm)**, mean ± SD | 30.6 ± 16.9 | 29.0 ± 18.7 | 0.868 |
| **Density (Calcified)**, n (%) | 2 (40.0) | 6 (40.0) | 1.000 |
| **Symptomatic Presentation**, n (%) | 2 (40.0) | 3 (20.0) | 0.582 |
| **Coexisting Lung Cancer**, n (%) | 1 (20.0) | 7 (46.7) | 0.596 |
| **Coronary Artery Disease**, n (%) | 4 (80.0) | 1 (6.7) | 0.005 |
| **Hypothyroidism**, n (%) | 3 (60.0) | 0 (0.0) | 0.009 |
| **Geographic Location**, n (%) |  |  | < 0.001 |
| Inland Provinces (Amasya, Corum, Tokat) | 5 (100.0) | 0 (0.0) |  |
| Coastal/Other (Samsun, Ordu, Baku) | 0 (0.0) | 15 (100.0) |  |

**Note:** SD = Standard Deviation. *p*-values are derived from the Mann-Whitney U test for continuous variables and Fisher's exact test for categorical variables.
